# ‘Are We Not Human?’ Stories of Stigma, Disability and HIV from Lusaka, Zambia and Their Implications for Access to Health Services

**DOI:** 10.1371/journal.pone.0127392

**Published:** 2015-06-03

**Authors:** Janet A. Parsons, Virginia A. Bond, Stephanie A. Nixon

**Affiliations:** 1 Applied Health Research Centre, Li Ka Shing Knowledge Institute, St. Michael’s Hospital, Toronto, Canada; 2 Department of Physical Therapy and International Centre for Disability and Rehabilitation, University of Toronto, Toronto, Canada; 3 ZAMBART, School of Medicine, University of Zambia, Lusaka, Zambia; 4 Department of Global Health and Development, Faculty of Public Health and Policy, London School of Hygiene and Tropical Medicine, London, United Kingdom; London School of Hygiene and Tropical Medicine, UNITED KINGDOM

## Abstract

**Background:**

The advent of anti-retroviral therapy (ART) in Southern Africa holds the promise of shifting the experience of HIV toward that of a manageable chronic condition. However, this potential can only be realized when persons living with HIV are able to access services without barriers, which can include stigma. Our qualitative study explored experiences of persons living with disabilities (PWD) in Lusaka, Zambia who became HIV-positive (PWD/HIV+).

**Methods and Findings:**

We conducted interviews with 32 participants (21 PWD/HIV+ and 11 key informants working in the fields of HIV and/or disability). Inductive thematic analysis of interview transcripts was informed by narrative theory. Participants’ accounts highlighted the central role of stigma experienced by PWD/HIV+, with stigmatizing attitudes closely linked to prevailing societal assumptions that PWD are asexual. Seeking diagnostic and treatment services for HIV was perceived as evidence of PWD being sexually active. Participants recounted that for PWD/HIV+, stigma was enacted in a variety of settings, including the queue for health services, their interactions with healthcare providers, and within their communities. Stigmatizing accounts told about PWD/HIV+ were described as having important consequences. Not only did participants recount stories of internalized stigma (with its damaging effects on self-perception), but also that negative experiences resulted in some PWD preferring to “die quietly at home” rather than being subjected to the stigmatizing gaze of others when attempting to access life-preserving ART. Participants recounted how experiences of stigma also affected their willingness to continue ART, their willingness to disclose their HIV status to others, as well as their social relations. However, participants also offered counter-stories, actively resisting stigmatizing accounts and portraying themselves as resilient and resourceful social actors.

**Conclusions:**

The study highlights a significant barrier to healthcare experienced by PWD/HIV+, with important implications for the future design and equitable delivery of HIV services in Zambia. Stigma importantly affects the abilities of PWD/HIV+ to manage their health conditions.

## Introduction

This paper focuses on experiences recounted by a group of Zambian persons participating in a study examining the intersectionality of HIV and disability[1–3]. The findings offered here concentrate on a specific feature of the data, namely the centrality of stigma in participants’ accounts. This stigma was told to have important effects on numerous aspects of their lives. The authors examine these data through the lens of narrative theory[4–7] to reveal how participants experience, internalize and resist stigma. Using an interpretive framework of story and counter-story[8], we highlight both how stigmatizing stories told about participants could result in real-world consequences, and also how their counter-stories served to resist such discourses. Participants suggested ways forward whereby those experiencing both disability (in various forms) and HIV can set their own places at the collective Zambian table[9].

### Context of the Study

The Sepo Study was initiated against a backdrop of growing recognition of HIV and disability as interconnected global health concerns[10]. In particular, the vulnerability of people with disabilities to HIV has received growing attention, with a focus on HIV prevention[11–14]. However, relatively little is known about the HIV care, treatment and support needs of people with disabilities who have become HIV-positive (PWD/HIV+)[15].

It is only relatively recently that the needs for HIV prevention and management strategies for PWD have been recognized [3,10,15]. While there has been renewed interest in HIV-related stigma in recent years [16–19], including in Southern African contexts [20,21], to date the experiences of PWD who later acquire HIV infection have not been explored in depth. Research (including that in Southern African settings) has also identified disability as a source of stigma in and of itself. A groundbreaking survey of 57 countries in 2004 conducted by Groce revealed that PWD are at increased risk on all known risk factors for HIV (e.g. poverty, sexual violence, lack of education) [11]. Despite the importance of this issue, prevalence studies of HIV amongst PWD are only now beginning to emerge [22]; for example, research regarding prevalence amongst deaf persons in Southern Africa reveals that they are at least as likely if not twice as likely to become infected with HIV as able-bodied persons [22]. The connection between *experiences* of HIV- and disability-related stigma has not been studied in depth and warrants further exploration.

The results reportedd here represent a subset of findings from the Sepo Study, which explored the experiences and perceptions of health equity issues for PWD/HIV+ living in Zambia (for example, access to HIV care, treatment and support), focusing specifically on the dual experiences of disability and HIV[1,2]. The Sepo Study involved interviews with PWD/HIV+ as well as key informants (KIs) working in the fields of HIV and disability. This paper focuses on important findings related to experiences of stigma shared by participants. The original intent of the broader study was neither to focus on stigma nor to offer a narrative approach to these data; nevertheless, an overarching theme of experienced stigma pervaded the dataset.

#### Informing theory: Using a narrative lens to illuminate experiences of stigma

For this paper we employ a narrative approach to the study’s findings in order to understand experiences of stigma amongst individuals living in particular circumstances and in a specific global context. Specifically, we draw on Frank and Nelson[4,6,8,23]. Both use narrative to explore the intersection of *self* and *other*, a space in which stigma is enacted in its most intimate form. A narrative constructionist stance is adopted, which assumes that identities are narratively constructed, socially situated and dialogical[4,8,24,25].

“Thinking with stories”, as Frank asserts[4,6], provides an important window into understanding the experiences of individuals and the meaning they assign to them[7,26]. Stories also connect tellers and listeners, and are a form of social action[27]. Stories are replete with moral messages, and can serve to portray for the listener “the type of person I am”[4]. Moreover, stories fulfill both descriptive and prescriptive functions, detailing what happened, but also what ought to happen next[5]. As such the narrators may have important solutions to offer.

Stigma has been described in many different contexts and with numerous definitions [18,28–30]. Stigma theory has shifted through three major frameworks[31]. Originally framed in terms of morality and religion over centuries, from the late 19^th^ century it shifted to a theory framed by sociological and psycho-social inquiry, with links to theories of deviance[32], social exclusion[29], and labeling [33,34]. A more recent framing is that of human rights, which has developed in response to a burgeoning body of literature on health-related (and specifically, HIV-related) stigma, and exposes social structural forces and political interests[18,35]. The latter two frameworks (sociological/interactionist and rights) can be seen as complementary[36]. The experiences of PWD who also are living with HIV within resource poor settings potentially stretch across all three frameworks.

The subset of data presented in this paper is best-suited to an interactionist framing. Goffman’s emphasis on the performative nature of social relations[29,37] informs our approach to these data, as the participants went to great lengths to describe how stigma was enacted in a variety of settings: from the clinical encounter, to the queue where they await care, to the community, and within their own families. Goffman noted at least two forms in which identity could be spoiled[29]: *discredited* identities are those where there is no hope of the person passing undetected during social relations, while *discreditable* identities are those that are not overtly detectable and where the stigmatized individual may attempt to pass as ‘normal’[29]. PWD with visible impairments may experience discredited stigma, and are vulnerable to being identified and labeled as different. As persons and as groups, they may be seen as outsiders by others [32,33]. Sartorius [38] identifies that the most intense experiences of stigma are when devaluation, exclusion and disadvantage occur together, meaning that PWD may be most vulnerable in resource poor settings. When PWD also become infected with HIV, the dimensions of stigma they can face extend and deepen. Their discredited identity (disabled) is compounded by their discreditable one (HIV+). Goffman’s typology of stigma fits well with the narrative stance adopted towards the data corpus in this study, since he frames stigma in the language of *relations* as opposed to attributes.

Adopting a narrative interactionist epistemological stance towards our data (and drawing on the *storied resource* and *dialogic* approaches to narrative identity outlined by Smith & Sparkes) encouraged us to see participants’ accounts as means by which they positioned themselves within their social environments[25]. Nelson argues that stories have important implications for identity configuration. Individuals may have damaging stories told about them, particularly in the case of one-sided stories where one person or group has unequal narrative, economic and/or institutional power[8]. Negative characterizations by others (including master narratives) can result in deprivation of opportunity for those they are about and can serve to constrain or control their ability to act in certain situations. Furthermore, these one-sided stories can become internalized and influence how people view themselves, what Nelson terms “infiltrated consciousness”[8](p.28), and which also has negative implications for agency and opportunity. However, Nelson also outlined how counter-stories can be told that challenge and resist such damaging stories; just as identities themselves are narratively constructed, they too can be re-shaped[8]. However, not just any story can be offered as evidence of a worthy identity in the face of stigmatizing accounts. Rather, counter-stories set out to shift the balance of power by challenging a “shared but oppressive understanding of who someone is”[8](p. 69). They offer narrative repair by permitting such persons to see themselves, and to be viewed by others, as worthy social actors [8,39].

## Materials and Methods

The study setting was Lusaka, the capital and largest city in Zambia. Lusaka is considered to be an HIV hyper-endemic setting with greater than 15% HIV prevalence among adults [40]. The prevalence of disability in Lusaka is difficult to determine; the highest estimate of disability prevalence (using a broad definition) in Zambia is 14.5% [41,42].

We used the definition of disability from the United Nations Convention on the Rights of Persons with Disabilities: *“*Persons with disabilities include those who have long-term physical, mental, intellectual or sensory impairments which in interaction with various barriers may hinder their full and effective participation in society on an equal basis with others*”* (Article 1)[43].

The study rationale and design were developed collaboratively with one of our Zambian research partners, a community-based disabled people’s organization called the Disability, HIV& AIDS Trust. Research ethics approval was received from the ethics review committees of three institutions: University of Zambia (Zambia), University of Toronto (Canada), and University of KwaZulu-Natal (South Africa). All participants gave informed consent. The majority gave written consent, but in a few instances verbal consent was obtained. Verbal consent was necessitated in some instances where participants’ impairments were such that they could not read the consent form (e.g. the participant had a visual impairment). Where verbal consent was obtained, this was documented by audio recording. This consent procedure was formally approved by all three ethics review committees.

### Participants

We recruited 21 people with disabilities who had become HIV-positive (PWD/HIV+), and 11 key informants (KIs) working in the fields of HIV and disability. Table 1 displays pertinent participant characteristics. Just over half of the participants with disabilities had mobility impairments. Participants were generally from a low socioeconomic status. Over half were women and all but one participant was on ART.

**Table 1:** Participant characteristics.

**Group 1. People with disabilities who had become HIV-positive+**		**21**
Age Range (years)		29–61
Sex		9 men, 12 women
Type of Impairment(s)	Hearing	3
	Mobility	12
	Visual	4
	Intellectual	1
	Mobility and intellectual	1
On ART at time of interview		20
**Group 2. Key informants working in fields of HIV and/or disability**		**11**
Type of organization	HIV community-based organization	2
	Disabled people’s organization	5
	Health services	2
	Government department	2
**Total # of participants**		**32**

The PWD/HIV+ were recruited in two phases (10 in Phase 1 and the remaining 11 in Phase 2) to allow for interim analysis and refinement of the interview guides in an iterative process. We used a combination of purposive and snowball sampling strategies[44] to interview women and men with a diverse range of impairments (i.e., physical, visual, hearing and intellectual). Snowball sampling was important for recruitment in this study since this is a highly marginalized population who are not visible in their communities. Therefore, creative means were required to identify participants in a sensitive way. Moreover, the principles underlying snowball sampling are consistent with the purposeful recruitment approach, whereby we sought participants with expertise and experience of being a person with a disability who is living with HIV. Participants had to be over 20 years old, and able to speak English, Bemba or Nyanja, three common languages in Zambia. Potential participants learned about the study through dissemination of recruitment information via posters and word-of-mouth within the HIV and disability communities in Lusaka. Potential study participants received study information verbally, in writing or in Braille from our research coordinator. Interviews were conducted primarily at participants’ homes, or at another convenient but private setting within the community.

All key informants were Zambians working in Zambia. For KIs, we purposively sought persons with experiences working in the areas of disability and/or HIV, including representatives from AIDS service organizations, disabled people’s organizations, HIV healthcare providers, as well as policy makers. Most were affiliated with disabled people’s organizations. Of note, several KIs also had mobility impairments and one was hearing impaired. Key informants were identified purposively using the networks of our research team. Potential participants were invited into the study by the Zambian Research Coordinator or the Principal Investigator (SAN). All interviews were conducted in English by a member of the fieldwork team in person except for one interview, which was conducted by the Principal Investigator by telephone. No compensation was provided for this group of participants (KIs) as we were advised by the Zambian members of our team that this incentive was not required.

### Data collection procedures

Data collection occurred between August 2010 and June 2011. The Zambia-based fieldwork team conducted all interviews and consisted of a project coordinator, seven fieldworkers (4 men and 3 women including PWD, sign language interpreters and HIV counselors) and two transcriptionists/translators. Fieldworkers were trained in qualitative interviewing techniques. In-depth, semi-structured 60–90 minute interviews were conducted in person, in Nyanja, Bemba, English or Zambian sign language. To ensure confidentiality, interviews were conducted at a location deemed sufficiently private, convenient and acceptable to participants. In two cases, interviews of KIs were conducted by telephone by the study lead (SAN). All interviews were digitally recorded and transcribed into English. Two transcriptionists/translators translated and transcribed all interviews. All transcriptions were quality checked for accuracy by the interviewers. Field notes were taken during and following each interview to record observational or contextual details not captured on the audiotape.

Our Zambian research coordinator matched participants with one of the six local, trained fieldworkers based on pragmatic (e.g., availability, locations) and cultural (e.g., age, sex, ability) considerations. Two of the fieldworkers were women with disabilities, two were HIV counselors, and two were certified Zambian sign language interpreters. All interviews with people with disabilities were conducted in person and in English, Nyanja, Bemba or Zambian sign language according to the preference of the participant. Interviews with participants who were deaf or hard-of-hearing were conducted in Zambian sign language with the fieldworker simultaneously verbalizing the comments of both the interviewer and participant to audio record the exchange. This process enhanced confidentiality by avoiding the need to engage a third person in the interview to interpret. Compensation was provided to the PWD participants.

The interview guide for PWD/HIV+ was designed to capture participants’ experiences regarding: (a) the intersectional nature of life with a disability and HIV; (b) issues of equity, inclusion and discrimination: (c) access to HIV care and treatment; and (d) government, NGO and community responses. Interviews with the KIs were designed to elicit their perspectives regarding the needs of PWD/HIV+, existing services, perceived obstacles (from individual to structural levels), and opportunities for improving health equity for this population.

Participants were assigned unique alphanumeric identifiers and personal details were removed to safeguard their identities.

### Data analysis

Data were analyzed using a collaborative, multi-phase process[45]. A subset of the larger Sepo Study project team first developed and piloted a coding framework based on concepts derived inductively from the interviews. Each transcript was then coded by two members of the research team and entered into a data organization software programme (NVivo 8.0). For the analysis in this paper, the lead authors reviewed specific nodes related to experiences of stigma. Further review of the transcripts was conducted using a narrative analytic approach[24,46–48], looking not only at content but also at the form and tone of stories told. A strategy of multiple readings of the transcripts was adopted[49], plus a constant comparative approach[48] to examine common themes and differences within and across interviews. We interrogated the dataset in terms of how interviewees positioned themselves in relation to reported events, in relation to others and in relation to themselves—not in static terms but as fluid, interactive and dialogic accounts [50]. Reflexivity was a priority and practice used throughout the study to enable the thoughtful engagement of our diverse team members. Epistemologically, we interpreted the interviews as a co-construction between interviewee and interviewer, adopting a social constructionist stance[51].

Techniques for ensuring analytic rigour included questioning, checking and theorizing[51]. By using multiple analysts with experience in different methodological approaches and theoretical traditions, alternative explanations were explored[51,52]. Importantly, authors sought to identify and consider the influence of their own social locations and experiences vis-à-vis life in Zambia, ability/disability, stigmatization and other planes of advantage/disadvantage throughout analysis. Other techniques for promoting rigour included accuracy checks, maintaining an audit trail of methodological decision-making, and discussing preliminary results with various knowledge users to ensure relevance and credibility[46,48,51–53].

## Results

Table 1 outlines the participants’ characteristics. Early in our analysis, it became apparent that the accounts offered by all participants highlighted the central role of stigma experienced by PWD/HIV+. Participants recounted how experiences of stigma in different settings had important consequences for their willingness and ability to seek HIV care and treatment [3] and obtain paid employment [54]. Using narrative analysis allowed us to interpret their accounts in a way that unified underlying themes in the data.

The following framework for organizing the stories offered by participants was used: accounts of how they perceived themselves, how others perceived them, how they internalized the accounts of others, and how they resisted stigmatizing (or othering) accounts. Below, we first present stories of self and other in three settings identified as important by participants: *stories of stigma in their clinical encounters* with health care providers (HCPs), *stories from the queue for HIV services* (on their way to receive care), and *stories of stigma within the community*. We then present *accounts of internalized stigma*. Finally, we present *counter-stories* offered by participants, which were interpreted as a way of resisting the damaging/stigmatizing stories told about them by others.

### Stories of stigma in the clinical encounter

Participants from both the PWD/HIV+ and KI groups offered numerous examples of stigma experienced during clinical encounters while seeking care and treatment for HIV. The negative experiences offered by participants were seen as directly contributing to negative health outcomes for PWD/HIV+. As one participant commented:

*I remember one cousin who was positive died because of stigma.*

(P2-01-PWD)

Many participants spoke about how they were seen as ‘other’ and ‘less than human’ by HCPs, both during testing for HIV and when seeking treatment. Becoming HIV-positive was seen as evidence that the PWD had engaged in sexual activity, which ran counter to prevailing assumptions that PWD are asexual. Further, living with disabilities meant it was harder to access information about HIV. As one participant commented:

*Some of this maybe is cultural or social beliefs around disability and sexuality*. *I think a lot of people don’t think that people with disabilities are sexual people*. *So*, *transmission can happen and sadly it’s too late*. *So there’s…not even an awareness that everybody needs [HIV prevention] information*

(P2-16-KI)

HCPs were described as commonly holding the same negative attitudes/stereotypes as the general population concerning the sexuality of disabled persons. Participants recounted that HCPs frequently expressed surprise when a PWD sought HIV care:

*…the doctor was surprised at me*. *He had laughed at me saying*, *“Can you also have HIV*?*” I then answered him that*, *“Doctor*, *I’m human*. *I also have the same feelings that you have*. *It’s only that the legs are the ones that differentiate us”…Even there in the room*, *they laughed*. *They didn’t treat me to say this person is also human*, *she can have*, *no*. *Until at last they tested me…But I had told them that…a lot of disabled people come to the hospitals*. *You discourage us…that’s not how it’s supposed to be*. *A disease that an able-bodied person can have*, *even me I can have it*.

(P1-07-PWD)

In this account, the participant experienced the additional burden of convincing HCPs of her need for help. Participants described sometimes needing positive test results to persuade HCPs that they were in the ‘right’ clinic.

This experience was not confined to PWD seeking HIV services. Similar stigmatizing encounters were recounted surrounding pregnancy and disability. This lends further support to the assertion that the negative experiences in the clinical encounter stem from commonly held assumptions regarding the sexuality of PWD and is one of the insights of stigma related to disability without the presence of HIV. One participant recounted:

*…sometimes even the medical personnel there*, *they say negative things about PWD*, *who are found in certain situations like having HIV*, *or if it’s for us women*, *if you are pregnant*, *and maybe it’s time for you to go for antenatal [care] or to give birth*. *Some of the comments that they make may make someone who is in that group too shy from accessing those services*, *and instead*, *die quietly in the home for fear that I’ll be victimized*.

(P2-26-KI)

The image of PWD preferring “to die quietly at home” rather than be subjected to the negative attitudes of HCPs is powerful. Here stigma is enacted in a space of care and with devastating consequences. The link between stigma and mortality and access to care acts as an urgent call upon the listener.

Participants told how such experiences of marginalization reinforce the sense that they are perceived differently from other patients seeking care. Participants repeatedly referred to being treated as “less than human”. The lesser status afforded to PWD was also exemplified by a KI who was an able-bodied HCP, who described PWD as follows:

*They are vulnerable people who need our support*, *who need our help*. *And more information should be given to them*, *to understand at their own level of learning…PWD are*, *they are just like young children*. *I mean in some other ways*, *I may be wrong*, *but young children who need very much help…*

(P2-19-KI)

Despite good intentions, this participant uses othering language (e.g. using “young children” to describe PWD). He later refers to PWD/HIV+ as “helpless”, which perpetuates stereotypes of victimhood[55]. Such accounts can be interpreted as a form of ambivalence towards working with PWD on equal footing.

While participants cited many instances where they felt marginalized during clinical encounters, they also provided rich and compelling descriptions of confronting the gaze of others while waiting in the queue for HIV services.

### Stories from the queue for HIV services

Participants who were PWD described their journeys to seek health services as difficult and disturbing. How they physically reached their local clinics depended partly on their impairments. For example, those experiencing blindness often required help from others to travel to the clinic, especially if vision loss was recent. Participants who were deaf were able to make their way independently. If they experienced mobility impairments, the need for assistance was related to the severity of the impairment. But the physical obstacles encountered on the way to accessing HIV services paled in comparison with the psychosocial burdens described.

One of the primary places where unwanted disclosure of HIV status was enacted was in clinic queues for HIV treatment. It was in these lines awaiting services that those with visible signs of disability were mocked and where othering began. As one participant recounted:

*…when one goes at the health center to collect those medicines*, *the people there…the so-called able-bodied*, *they pass comments*, *“Look at that one*. *He’s disabled*, *at the same time HIV-positive*. *He doesn’t even feel sorry for himself*. *If it was me…I wish maybe I die”*.

(P2-07-PWD)

Several PWD/HIV+ described the uncomfortable feeling of being singled out because of their visible impairments.

*When you go to collect medicine*, *they stare a lot at you*. *When they call your name*, *they see you getting up with a walking stick*, *they stare at you until you get in…every time I’m walking like that*, *I tend to think that they obviously remain talking about me*. *Since in a situation where you’re a lot*, *maybe 100 [people]*, *and I’m the only disabled*.

(P2-08-PWD)

As occurred in their accounts of interacting with HCPs, such stigmatizing experiences in the queue can have important health consequences. Again, people told of considering foregoing ART rather than being subjected to the taunts of others:

*On the queue again*, *your friends [are] laughing at you to an extent of not even going there sometimes*, *thinking*, *“Ah*! *That day they laughed at me*, *today I should just stay [home]”*. *They do laugh*, *“Eh*, *even the disabled are found here*?*” Sometimes therefore you feel shy*. *Let me just stay home*.

*(P2-13-PWD)*

Because everyone must queue up to access HIV services, this amounts to a public ‘outing’ for PWD as sexual persons, which runs counter to the prevailing discourse of asexuality surrounding disability. In addition to stigma related to HIV generally[16,20], people with visible impairments appear to experience a double burden of stigma by virtue of their status as disabled. The implication that having a double burden is too much reflects in part a limited capacity to cope in the wider precarious context of many poorer Zambians. In the pecking order of the queue, able-bodied persons seeking HIV services may be seen as telling damaging stories about PWD/HIV+ in order to demonstrate that they are like everyone else.

Some PWD/HIV+ also noted that the public setting of the clinic queue meant that passersby could see them seeking HIV care, leading to disclosure within the community. As one participant stated:

*… there’s one neighbour who saw me when I went to get medicine … it seems like she publishes the information*. *Nowadays I don’t even sit outside to chat*. *I feel that when they are sitting there*, *obviously it’s me they are talking about*.

(P2-11-PWD)

This suggests that the act of seeking care presents a special danger for PWD in terms of their social identity. The gossip that ensued once participants were identified as seeking HIV services was described as spilling out into the social world beyond the clinic. In this way, they told how their identities became discreditable, under the gaze of others.

### Stories of stigma within the community

Beyond the clinical encounter and the queue for HIV services, participants also described negative attitudes from those within the broader community, including their social circles of family and friends. Their accounts indicate that for PWD/HIV+, stigma pervades most aspects of their lives.

Once their HIV status was known to others within the community, participants told of encountering very negative attitudes. One PWD recounted:

*I and my spouse are members of the [disabled people’s organization]*. *So people think that maybe my wife is a prostitute [because she is HIV+]*. *I don’t think she is a prostitute*. *I have kept this information to myself*. *There is too much stigma saying that ‘Have you seen that one sitting there*? *He is positive*. *He is HIV-positive*.’

(P2-01-PWD)

This is a further instance of othering talk whereby the person who contracted HIV is assumed to be engaging in what is perceived to be improper sexual behavior (colloquially referred to as ‘prostitution’), and that this is the explanation offered for why PWD might become HIV +. They are other from those who engage in this talk. Participants repeatedly offered instances where they felt that others saw them as not human.

*So they keep a distance from you like that*, *they don’t count you as a person when they see that you’re HIV-positive and then you’re disabled*. *They begin gossiping about you*, *even the neighbours*.

*(P2-15-PWD)*

Disclosure as HIV-positive also had implications for participants’ relations with their families, with some reporting a lack of support from family members (for child rearing, employment). A participant cited estrangement from family members resulting from HIV diagnosis as follows:

*Now my blood brother is a thorn in my flesh*. *He despises me and…he has never visited me from the time he heard about that [HIV]*, *even when I send letters saying please come and pay me a visit*.

*(P2-01-PWD)*.

Evidence of othering experienced in the community included participants overhearing able-bodied people claim they (PWD/HIV+) were ‘better off dead’.

*No*, *we don’t feel good because what they normally say is offending*. *They say*, *“This person*, *she is disabled and she adds on this [HIV] disease…*.*the doctor that gives her medication*, *if only he could give her one for killing her*.*”*

*(P2-11-PWD)*

These findings illustrate the ways in which HIV and disability-related stigma was experienced not only in the clinic setting but also throughout participants’ wider communities. The stigmatizing attitudes of others had implications not only for their social relations with family, friends and community members, but it also affected their opportunities to make a living. While work experiences of PWD/HIV+ are the topic of another paper [54], it is important to mention here that many of the participants recounted finding most, if not all, avenues for earning a living closed to them once their HIV status became known. Many resorted to begging because no other work was available. Their accounts offer tangible and extreme examples of deprivation of opportunity.

### Accounts of internalized stigma

Surrounded as they were by this othering talk—in the clinical encounter, in the queue, in the broader community—it is unsurprising that some participants appeared to internalize stigmatizing attitudes in their accounts. One participant with a mobility impairment commented,

*Things have changed*. *When I’m walking*, *I do despise myself sometimes*. *I despise myself*.

*(P2-13-PWD)*

Such negative self-perceptions can have important consequences, including suicidal thoughts:

*…it was very difficult for me with those able-bodied people*, *because you know*, *my situation being a disabled and also getting HIV/AIDS*. *If someone laughs at me*, *it didn’t feel as if I was a person*. *I even came to the extent sometime to…feel that I should just get out of this world and maybe be a dead person*, *so that they don’t intimidate me in this way*.

*(P2-10-PWD)*

Here we have come full-circle, whereby the reluctance to attend clinic occurs when the PWD/HIV+ becomes other to themselves, internalizing the prevailing attitudes, until they find the burden of resistance too great. Resigning from the clinic may be one form of internalized stigma, but there are other ways in which PWD/HIV+ take up othering attitudes. One participant recounted blaming himself for becoming HIV-positive:

*I’m regretting because*, *what caused you to be in that state*?*…I was the one doing it*, *yes*. *Going into taverns*, *doing whatever…*

*(P2-07-PWD)*

Another participant summed up this experience eloquently:

*The problem which [we] sometimes face was the stigma we had amongst ourselves…amongst we PWD*. *Because some were thinking a disabled person cannot be infected with the virus*. *And that myth has carried a lot of us in*, *they have been buried today and they have died because all they’ve been thinking to say was HIV is for people who are able to do and… for example those who are sex workers*.

*(P2-14-PWD)*

These participants’ accounts demonstrate how some internalize the negative attitudes encountered both inside and outside clinics. They talked about how hard it was to accept their own HIV diagnosis when they were already disabled. This uptake by PWD/HIV+ of the prevailing societal master narrative could be interpreted as an example of infiltrated consciousness, as described by Nelson.

However, within a single interview, such accounts are often paired with competing narratives that resist the negative accounts imposed (from within and without). These counter-stories serve to resist the damaging stories told about them by others and to construct a positive identity for themselves[8,56].

### Counter-stories: resisting the stigmatizing master narrative

While participants described an often stigmatizing social terrain, we were struck by how accounts of negative othering were often juxtaposed with commentary demonstrating resistance to these attitudes. While participants described being treated as less than human, they also used their stories to portray themselves as worthy social actors[39,56].

*I have my inherent rights and we need to be on the same level with others*. *Those that are [HIV]-positive and negative*, *they are all human*. *For instance*, *when we are in the workshop we are all learning whether one is [HIV]-negative or positive*. *There is a right for us to be equal*.

(P2-01-PWD)

Both able-bodied and disabled persons with HIV were sometimes integrated within these workshops focused on managing HIV. Such settings were described by PWD as opportunities for interacting in positive ways with their able-bodied counterparts. This use of the able-bodied person living with HIV as a way to seek acceptance was offered in a number of accounts, as if to emphasize for the listener the shared experiences of able-bodied and disabled persons alike. If both can contract HIV, how different can PWD be? An opportunity to dissolve the distinction between self and other presents itself.

Such positive stories counteract the prevailing master narrative of spoiled identity[29]experienced by the PWD in our study, and demonstrate opportunities to reach out to others within the community. While some able-bodied persons with HIV might stigmatize them, instances of respect were offered:

*…*. *there are able-bodied people but with HIV/AIDS*. *Some are…even admiring the situation that I’m in*. *Like*, *I’m renting a home*, *whereby I’m only a woman*, *with HIV/AIDS and with a disability*. *But I could [do] everything*. *Today I could do everything rather than someone who’s able-bodied and has HIV/AIDS*. *They are always complaining…I even encourage them to say*, *what of me who’s disabled and HIV/AIDS*? *I’m your example…“What of my sister who’s a disabled person and has got HIV/AIDS*? *What of me*? *I have only HIV/AIDS*, *I’m not disabled*.*”Really*, *I do encourage many of them in the community*.

(P2-10-PWD)

By portraying herself as a positive role model, this participant demonstrates how she can be an inspiration to both able-bodied persons living with HIV as well as PWD. This embracing of a positive identity, not only as a fellow human being, but one who extends herself by offering compassion to others in need was demonstrated in numerous counter-stories from participants. These counter-stories serve as more than a buffer against stigmatizing attitudes; they provide opportunities for demonstrating agency and generosity[4] in the face of highly negative attitudes, although they also simultaneously carry the burden of working harder. The counter-stories act as a leveling device, demonstrating that some PWD/HIV+ are accepted and actively working to combat stigma and misunderstanding.

*With my family members who have not understood my situation*, *I always emphasize to them*, *“Look*, *being a human being*, *I have to face a lot of challenges*. *And one of the challenges is HIV/AIDS*, *which is a pandemic in every part of the world*. *And I cannot be spared that because*, *being a human*, *I pass through frustrations*, *through peer pressure and everything like that”…How my infection came*, *to them*, *it does not matter*. *All what matters is the shame they have found in me*. *So I always emphasize to them*, *“At least me with or without the virus*, *I’ll still be the same person you knew before*. *I’ll still not change*. *I’ll be disabled*, *but…I have to carry on with my status so that…if one of you may come in a situation I’m in*, *I’ll be able to understand you and maybe even giving you words of encouragement*.*”*

(P2-14-PWD)

This is the language of courage and serves as a message to those in her social circle that her family members are not alone, but that there is an opportunity of belonging together[57,58].

The KIs who were also PWD not only drew on their experiences of providing services to PWD in a broader context, but likewise drew on their own experiences of being stigmatized. A KI with experiences of disability commented:

*What the community should know is we are human beings*. *The fact that we have disabilities does not make us maybe special or does not make us not have feelings*. *Because if we can eat*, *if we can cry*, *then we can also have sex*. *And if we can have sex*, *we can have the consequences of sex*. *It’s either a pregnancy or HIV/AIDS*, *if it’s not protected…But the response*, *like what I’ve known personally*, *it’s not good*. *Yeah the communities…sometimes they even become violent*, *especially the family*!

(P2-26-KI)

As a PWD, she challenges the prevailing master narrative concerning the presumed asexuality of PWD, arguing instead for recognition of the common humanity between able-bodied and non-able-bodied persons. She also exposes a pattern of violence which women and other vulnerable groups are more likely to experience.

Another form of counter-story uses the language of human rights to resist the kind of discrimination participants recounted. With respect to access to medical care one PWD/HIV+ person stated:

*I can say as a deaf [person] who has got a virus and then someone who is able-bodied*, *we have the same right even accessing medical support*. *It is my right as a deaf person to have a sign language interpreter in hospital for good communication … As a deaf person if I meet a medical staff it is very difficult for me to access information*.

(P2-01-PWD).

This offers HCPs constructive advice and a way forward for enhancing the quality of care provided to PWD/HIV+.

The participants’ counter-stories serve the function of advising the listener on ‘what ought to happen’[5] and offer moral arguments for why this situation must change[8]. They also offer examples of what is working currently, and this has to do with recognition of PWD as familiar, not other[57]. The counter-stories offer the connection of common humanity between PWD and their able-bodied counterparts[59].

*…some people this side [of the clinic] have started knowing me*. *At times when I’m late*, *they squeeze me a little[in the queue]*. *They put me somewhere in front*, *so that I can go fast*. *That’s what has caused me not to leave*, *because they’ve started getting used to me…I’ve started getting used to those people… they know you*, *and will greet you*. *Now with new people*, *maybe you’ll be there just alone as though you are lost*.

(P2-08-PWD)

This recognition is not simply an abstraction, but translates into real-world consequences for PWD, altering their lived experiences of the queue and medical care. But the transient and idiosyncratic quality of this recognition is likewise captured in this account. If you end up on the ‘wrong side’ of the clinic, you are back to being marginalized, confronting the master narrative yet again. The wording “you are lost” speaks to the profound impact lack of recognition has, with implications for identity as well as care provision.

Finally, both PWD/HIV+ and KI participants spoke about the power/importance of PWD supporting one another and being agents of change in their own lives.

*…*.*at least we [can] have our own centers*, *as disabled people*. *There*, *things will be easy for us … We need our own centers where we can get medicine freely…maybe if we fight for a center where we can get the medicine*, *those people who will come after us*, *they’ll be using the same center*.

*(P2-07-PWD)*

Taken together, the counter-stories offered by participants can be interpreted as accounts of resisting the negative stories told about them by able-bodied persons. They indicate that PWD/HIV+ can and do portray themselves positively and as fellow citizens deserving of care and respect. They also point to concrete ways in which conditions for PWD/HIV+ can be improved, and they frame themselves as agents of change. Their accounts are aimed at PWD/HIV+ but in telling them they are not only addressing themselves but also others who are outside their group. The counter-stories offer resistance by repudiating and contesting the master narrative [8].

## Discussion

This paper has examined accounts of stigma and marginalization as experienced by a group of Zambian participants, most of whom were PWD who had become HIV-positive. Participants recounted that for PWD/HIV+, stigma was enacted in a variety of settings, including the queue for treatment services, their interactions with HCPs, and within their communities. Stigmatizing accounts told about PWD/HIV+ were described as having important real-world effects. Not only did PWD/HIV+ recount stories of internalized stigma (with its damaging effects on self-perception), but that negative experiences also resulted in some PWD preferring to “die quietly at home” (P2-26-KI) rather than being subjected to the stigmatizing gaze of others when attempting to access life-preserving ART. However, participants also offered counter-stories, actively resisting the stigmatizing master narrative and portraying themselves as resilient and resourceful social actors. It was evident that disability stigma dovetailed with HIV stigma, demonstrating how vulnerabilities can become complementary and stigma can be layered[17,60]. However it was also evident that the ability to rise above the disadvantage of disability could emblazon some PWD with resilient and affirmative strategies.

Examining participants’ accounts through the lens of narrative theory[5,6] illuminates how the negative attitudes of others were encountered in many settings and that stigma and marginalization are enacted in their everyday social relations (not just during clinical encounters), and can result in deprivation of opportunity and constrained agency[8]. The stories offered here reveal how stigma shapes participants’ self-perceptions. The interviews can be seen as contextually-situated performances in which participants enact their identities[61]. Narrative constructionist approaches matter precisely because of the centrality placed upon the interaction of self and other[8,24]. Bamberg emphasizes that we use narratives to make sense to ourselves but also to others (including generalized others in the wider cultural realm), and as such narratives are socially situated[62]. Narratives and counter-narratives are also fluid and evolving over time—not fixed, but open to re/interpretation [62]. The participants in our study could be considered to be engaging with master narratives, which Bamberg defines as general cultural expectations or “‘frames’ according to which courses of events can be easily plotted, simply because one’s audience is taken to ‘know’ and accept these courses.” [62](p.366) The hegemonic master narrative of PWD being asexual can serve to constrain the agency of individuals, but the participants’ contest this through their counter-stories, which can be interpreted as attempts at reclaiming agency and opening up alternative understandings for the listener[62].

While many of the accounts offered by participants are disturbing in the extent to which PWD/HIV+ are stigmatized, it seemed particularly troubling that stigmatizing attitudes pervaded their accounts of interacting with HCPs. Why would it be seen as acceptable for HCPs to treat PWDs in this way? One possible explanation is that PWD are seen as wholly other by the HCPs, that they adopt the prevailing master narrative concerning the sexuality of PWD. However, othering is a complex process and other complementary explanations are possible. For instance, Frank’s notion of *narrative habitus* may offer a more nuanced interpretation, in that HCPs could be thought of as predisposed towards certain stories concerning PWD. Frank asserts that “narrative habitus is the embedding of stories on bodies” [23](p. 52); the HIV status of the PWD they care for runs counter to the HCPs’ perceptions of disabled bodies as desexualized, and are therefore perceived as troubling. Or it may not yet be within the HCPs’ *repertoire* of recognizable stories[23]. But the repertoire and the habitus are not fixed and unchangeable[23]. The counter-stories provided by the participants in this study are framed to counteract this othering stance, and could be interpreted as (potentially) disrupting the narrative habitus of the HCPs, offering new stories for their “inner libraries”[23]. How do such stories work to challenge damaging discourses and shift the balance of power in these clinical encounters? We would suggest that they could be used to ignite the “moral imagination” of HCPs, which Momeyer and others suggest is important for understanding the perspectives of others from their own standpoints and for seeing alternative solutions as possible[63]. Their counter-stories may also expand the “sense of life’s possibilities”[23] (p. 54) both for the PWD/HIV+ who tell them and for those who will listen.

The term ‘stigma’ has a long history in health services research[18,28–30,64]. Goffman commented that it is in “mixed” social situations, in the relational space between the stigmatized and the unstigmatized, where feelings of uncertainty and fear of unwanted disclosure (in those perceived to be discreditable) become foregrounded[29]. This was described in eloquent detail by our participants who experienced what was at once a “discredited” identity (having a disability visibly detectable to others) and a “discreditable” identity (HIV, which if undisclosed, might allow one to pass undetected)[29]. Their stories and counter-stories reveal an ongoing re/negotiation of their identities that continues to unfold [65]. HIV-related stigma has received considerable attention[18,21,66]. Accounts of stigma offered here contribute further to this literature by indicating that stigma is experienced by PWD/HIV+ in their interactions with others, with significant implications for their relations with family, neighbours, and employers.

Participants recounted that seeking HIV services frequently functioned to ‘out’ them, whereby their HIV status (and presumed sexual activity) was disclosed to the broader community while queuing for care. This is similar to the findings of Bond and Nyblade (2006) whereby Zambian public health measures such as the use of TB corners function to disclose both a person’s TB and HIV status[21]. Even among those who are able-bodied, testing is fraught with dangers and threats to one’s social identity. Because of the public nature of clinics, and the social consequences if one’s status becomes known, people may delay testing. Bond (2010) comments that the decision to test is “often a protracted, courageous and painful decision”[20](p. 8). With HIV, perceived culpability is considerably heightened by the links between HIV and so-called improper sexual behaviour[60], as well as by the links with death and the ability of HIV to either be concealed or lead to physical frailty. Given the high prevalence of HIV generally in Zambia (15%) and many other countries in Southern Africa, stigma attending HIV infection in the absence of disability remains signficant[20]. Bond highlights a reluctance to be tested for HIV among Zambians generally (20% of men and 35% of women), with this silence stemming directly from HIV-related stigma[20]. HIV-related stigma contributes to greatly curtailed opportunities for HIV+ persons, including lower incomes and employment opportunities [67],

A systematic review on disability and HIV in Southern Africa by Hanass-Hancock indicates that disability itself carries its own stigma[13]. Although Zambian studies were not included, the data from the 11 countries included indicate that PWD are less likely to have access to education as children and are more likely to be orphaned (secondary to desertion by their parents)[13]. PWD tend to be the poorest members of their communities, with curtailed opportunities for making a livelihood for themselves [22]. PWD are also more likely to be subjected to sexual abuse and exploitation [13], coupled with much poorer access to legal protection [22]. This is compounded by misconceptions that sex with a virgin or a disabled person can cure HIV [13]. In another paper written by the Sepo team concerning the work experiences of PWD/HIV+ [54], we highlight that opportunities for making a living are greatly curtailed for this population, even more so than is usually experienced by PWD living in Zambia.

The real-world implications of our study’s findings for the delivery of health care and HIV management to PWD should not be underestimated. If PWD/HIV+ are reluctant to access life-preserving ART because of fears of stigmatization from HCPs (and others), then it seems very likely that this will translate into lower survival rates for this population. At present, it is hard to get an accurate picture of true survival rates in this population, given that PWD may not disclose their HIV status and may die unseen and unheard, as so eloquently recounted by participants in our study. To date there have been relatively few studies undertaken in Zambia to capture rates of survival from HIV infection amongst PWD. Similar gaps in our knowledge exist related to known transmission rates amongst PWD in this context. However, there is literature to suggest that PWD are particularly vulnerable to HIV transmission, since virtually all known risk factors for HIV infection are elevated amongst PWD [22]. Of particular concern is the lack of HIV prevention and sexual education resources for PWD in many countries, including those of Southern Africa[13,22].

In our study, not only was stigma enacted through their relations with others, but the master narrative was also incorporated into how PWD view themselves, and can be interpreted in Nelson’s terms as ‘infiltrated consciousness’[8]. Despite this, they also manage to resist stigmatizing stories told about them by offering counter-stories that allow them to portray themselves in ways that open up opportunities for recognizing the common humanity shared between teller and listener[5,59], and to construct identities of which they can be proud (e.g., as agents of change, generous fellow citizens, hard workers)[39]. The counter-stories allow participants to trade a master narrative of spoiled identity for a more positive one and a restoration of agency[8,20,29].

These counter-stories are remarkable in that they indicate resilience in the face of overwhelming stigmatization. We are not suggesting that this additional burden of going above and beyond to prove one’s moral worth should be placed upon PWD/HIV+. While the resistance to stigmatizing accounts portrayed by our participants is admirable, as Howarth asserts, resisting stigma is “a collective enterprise” [55](p. 449). Any change must be relevant to the context in which stigma is enacted, and based upon community resources and collective will[55].

This study had a number of limitations. First, the study relies heavily on the researchers’ interpretation of accounts of participants speaking four different languages. Different theoretical and methodological lenses could lead to different interpretations. Nevertheless, the narrative stance taken for this analysis serves to illuminate experiences of profound stigma and its implications for PWD/HIV+; it also serves to demonstrate how PWD in this context resist damaging stories told about them. Moreover, two of the researchers reside in a very different cultural context (Canada), although this is mediated by one co-author living in Zambia. In addition, our sample was over-representative of those with mobility impairments, while few had intellectual impairments. Greater efforts to include the perspectives with persons with intellectual impairments should be a priority in future research. We also note that we did not study the social structural forces underlying such stigma experiences. Taking a narrative stance towards this data subset allowed us to explore enacted and felt stigma in meaningful ways [64]. However, we agree with Scambler that stigma relations are embedded within broader social structures, and are ultimately related to issues of power and economy[64]. These should be a focus for future research and could include comparison between PWD of different socio-economic status and how this might influence the stigma they face. Finally, a stronger focus on how PWD experience stigma without and with HIV might help disentangle further the relationship between these different stigmas.

### Conclusions

The advent of ART in Southern Africa holds the promise of making the experience of HIV approximate that of living with a chronic condition. However, this potential can only be realized when all persons living with HIV are given equitable access to care. The experiences outlined by our participants indicate that stigma importantly affects the ways in which PWD/HIV+ are able to manage their health conditions. As we and other researchers have shown, stigmatized individuals are more likely to feel ashamed, hide their HIV status, and discontinue ART, which may not only result in tragic consequences for the individual, but a greater likelihood of transmission of HIV into the broader community (including drug-resistant strains)[20]. At the practice level, stigmatization has implications for access to healthcare services, quality of life and, ultimately, survival[18]. While PWD directly experience stigma, the consequences go beyond these individuals into wider society. At a policy level, the global health community’s goal of zero new HIV infections and zero HIV-related deaths will be difficult to achieve without addressing the constraints on care encountered by PWD/HIV+.

Levinas argued for the importance of recognizing the sovereignty and dignity of individuals while recognizing our connections and obligations to them[57]. He asserted that it becomes ‘permissible’ for individuals to treat other people badly when they are seen as wholly other to themselves, and when they fail to recognize the common humanity between themselves and others. This is not unique to the Zambian context, but is a recurrent theme through the history of humanity and occurs as commonly in high-income as low-income settings. Conversely, once we recognize the other in ourselves, we have a moral obligation to them. The counter-stories offered by the participants in this study serve as such a moral hailing[4] to able-bodied persons to see PWD as equals.
